# Analysis of Phospholipids, Lysophospholipids, and Their Linked Fatty Acyl Chains in Yellow Lupin Seeds (*Lupinus luteus* L.) by Liquid Chromatography and Tandem Mass Spectrometry

**DOI:** 10.3390/molecules25040805

**Published:** 2020-02-13

**Authors:** Cosima Damiana Calvano, Mariachiara Bianco, Giovanni Ventura, Ilario Losito, Francesco Palmisano, Tommaso R.I. Cataldi

**Affiliations:** 1 Centro Interdipartimentale SMART, Università degli Studi di Bari Aldo Moro, via Orabona 4, 70126 Bari, Italy; ilario.losito@uniba.it (I.L.); francesco.palmisano@uniba.it (F.P.); 2 Dipartimento di Farmacia- Scienze del Farmaco, Università degli Studi di Bari Aldo Moro, via Orabona 4, 70126 Bari, Italy; 3 Dipartimento di Chimica, Università degli Studi di Bari Aldo Moro, via Orabona 4, 70126 Bari, Italy; mariachiarabianco@yahoo.it (M.B.); giovanni.ventura@uniba.it (G.V.)

**Keywords:** LC-ESI-tandem MS, food, phospholipids, fatty acids, *Lupinus luteus*

## Abstract

Hydrophilic interaction liquid chromatography (HILIC) and electrospray ionization (ESI) coupled to either Fourier-transform (FT) orbital-trap or linear ion-trap tandem mass spectrometry (LIT-MS/MS) was used to characterize the phospholipidome of yellow lupin (*Lupinus luteus*) seeds. Phosphatidylcholines (PC) were the most abundant species (41 ± 6%), which were followed by lyso-forms LPC (30 ± 11%), phosphatidylethanolamines (PE, 13 ± 4%), phosphatidylglycerols (PG, 5.1 ± 1.7%), phosphatidic acids (PA, 4.9 ± 1.8%), phosphatidylinositols (PI, 4.7 ± 1.1%), and LPE (1.2 ± 0.5%). The occurrence of both isomeric forms of several LPC and LPE was inferred by a well-defined fragmentation pattern observed in negative ion mode. An unprecedented characterization of more than 200 polar lipids including 52 PC, 42 PE, 42 PA, 35 PG, 16 LPC, 13 LPE, and 10 PI, is reported. The most abundant fatty acids (FA) as esterified acyl chains in PL were 18:1 (oleic), 18:2 (linoleic), 16:0 (palmitic), and 18:3 (linolenic) with relatively high contents of long fatty acyl chains such as 22:0 (behenic), 24:0 (lignoceric), 20:1 (gondoic), and 22:1 (erucic). Their occurrence was confirmed by reversed-phase (RP) LC-ESI-FTMS analysis of a chemically hydrolyzed sample extract in acid conditions at 100 °C for 45 min.

## 1. Introduction

Lupin seeds are fruits of an annual herbaceous plant belonging to Fabaceae family of the genus *Lupinus*, species albus. There are 12 lupin species native to Europe and Mediterranean regions. Two of them, namely the European white lupin or *Lupinus albus* and the yellow lupin or *L. luteus* [1], are extensively farmed. Edible lupins are usually cooked and stored in salts to lower the content of alkaloids. Lupin seeds are used to replace cereals or soy in baked goods or pasta, as they are gluten-free and are suitable for people with celiac disease [2]. Apparently, lupin seeds possess beneficial properties and can be considered as a functional or nutraceutical food, which are able to decrease the glycemic index and play a key role against obesity [3], diabetes, and heart diseases [4]. Lupin seeds are rich in arginine along with all essential amino acids [5] and lead to cholesterol reduction thanks to the high fiber content, which also improves intestinal functions. 

To correlate the beneficial effects of lupin components, many studies have been carried out by largely focusing on the characterization of protein [6] and vitamin content [7,8]. With regard to lipid fraction, the ratio content between polyunsaturated and saturated fatty acids (PUFA/SFA) in lupin seeds falls in the range of 1.3 to 2.9:1, which is significantly higher when compared to meat-based foods [9]. High PUFA/SFA ratios represent an additional feature since the relatively low content of SFA contributes to prevent coronary heart diseases [10]. Most fatty acyl chains are normally comprised into phospholipids (PL), which are recognized as minor bioactive constituents exerting healthy effects as antioxidants including those with free amino groups such as phosphatidylethanolamines (PE). Since dietary PL deliver their fatty acids (FA) for incorporation into cellular membranes, it is of paramount importance to know the PL composition of lupin seeds by also considering the great impact on the membrane fluidity and lipid rafts formation [11].

As far as we know, the characterization of major PL has been afforded in white lupin *Luteus albus* by thin-layer chromatography (TLC) [12,13]. Although all PL spots were identified by reference compounds, the quantitative determination of phosphorus was carried out by colorimetric analysis. The most abundant class is given by phosphatidylcholines (PC), which is followed by phosphatidylethanolamines (PE) and phosphatidylserines (PS). Minor contents of phosphatidylinositols (PI), phosphatidic acids (PA), phosphatidylglycerols (PG), and lysoPC (LPC) were reported as well [12,13]. 

Liquid chromatography-mass spectrometry in conjunction with electrospray ionization (LC/ESI–MS) is the preferred approach to identify phospholipids in complex mixtures of lipid extracts [14]. Based on our knowledge, a complete structural characterization of intact PL in yellow lupins, especially in terms of their esterified fatty acyl chains, is missing. In this work, hydrophilic interaction liquid chromatography (HILIC) with ESI coupled to either high resolution/accuracy Fourier-transform orbital-trap MS (HILIC-ESI-FTMS) or linear ion-trap (LIT) tandem mass spectrometry (HILIC-ESI-MS/MS) was applied to investigate the phospholipidome of yellow lupin seeds. In recent years, amphiphilic compounds such as phospholipids have been successfully separated by HILIC [15,16,17] using a silica-based stationary phase and an organic aqueous mobile phase in gradient elution [18]. The chromatographic run is accomplished with high percentages of acetonitrile (ca. 80% *v*/*v*) in the mobile phase. Thus, an efficient desolvation and ionization of compounds in the electrospray (ESI) source are guaranteed [19]. Moreover, a separation based on the head group polarity leads to simplified chromatograms of very complex mixtures with each peak/band almost corresponding to a specific PL class.

The ESI source in a negative ion mode was selected to be more informative than positive ionization. The combination of accurate MS and tandem MS measurements allowed us to identify more than 200 PL including lyso-PL (LPL). The distribution of linked fatty acyl chains was found to differ among PL as palmitic, oleic, and linoleic are the most abundant in PI, PG, PE, PC, and LPC and polyunsaturated linoleic and linolenic predominant in PA. The identification of these fatty acyl chains was also obtained upon chemical hydrolysis in acidic conditions of the sample extract. Reversed-phase liquid chromatography coupled to ESI-FTMS was applied to survey the in-vitro formed FA from digested PL/LPL, as recently reported for mussel samples [20]. The relative distribution of FA from various analyzed samples confirmed that oleic acid was the most abundant, which is followed by linoleic, linolenic, palmitic, erucic, and gondoic acids. The PUFA + MUFA/SFA ratio was around 5.0 ± 0.5 on average, which confirms the remarkable nutritional value of yellow lupin seeds.

## 2. Results and Discussion

### 2.1. Phospholipid Analysis of L. luteus Seed Extracts by LC-ESI-FTMS

**Figure 1:**
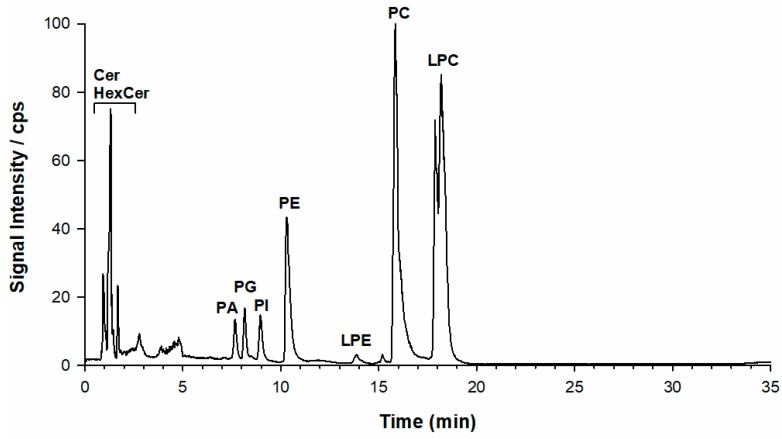
Representative HILIC total ion chromatogram (TIC) of a lipid extract of *L. Luteus* seeds (see Experimental and text for further details). Legend for PL classes: PA phosphatidic acids, PG phosphatidylglycerols, PI phosphatidylinositols, PE phosphatidylethanolamines, LPE lysophosphatidylethanolamines, PC phosphatidylcholines, and LPC lysophosphatidylcholines.

Figure 1 shows a representative total ion current (TIC) chromatogram in a negative ion mode of a sample extract of *L. luteus* seeds after solid phase extraction (SPE) (see Experimental section). All lipid classes were separated in less than 20 min with the elution order reflecting the increasing polarity of the headgroup, i.e., PA > PG > PI > PE > LPE > PC and LPC [21], as retrieved using external standard compounds. As expected, lyso-forms bearing a single acyl chain were more retained than the corresponding PL classes [22]. The chromatographic profile exhibited a separation between PC and two main LPC bands with the elution order depending on their regiochemistry (*vide infra*). The negative ESI mode of lipid species was chosen for being more informative with a better signal-to-noise ratio than a positive ionization one [23]. Based on the chemical structure of PC and LPC, these species were detected in a negative ion mode as formate, acetate, or demethylated adducts. A preliminary source-induced fragmentation (sid) was applied at a collisional energy of 40 eV to enhance the generation of demethylated [M − CH_3_]^−^ ion diagnostic of the choline polar head. A snapshot about the lipid composition of a given PL class was obtained by looking at the MS spectrum averaged over each HILIC band. Figure S1 (Supporting Information) is displayed as the ESI(−)-FT mass spectra relevant to PA (A), PG (B), PI (C), PE (D), PC (E), and LPC (F) averaged under each chromatographic band of the lipidic seed extract. A preliminary PL identification was achieved by using the freely available online lipid calculator tool by searching for accurate *m*/*z* ratios of FTMS data (Figure S1), which sets a tolerance of ±0.005 *m*/*z*. We anticipated that the HILIC separation was very successful for the identification of PL and the proper designation of isobaric species (see below). A list of all peaks extracted from averaged spectra were screened against a library of reference species and only *m*/*z* values which exhibits that accuracies lower than 5 ppm were accepted. 

### 2.2. CID-MS/MS of Phospholipids in the Lipid Extract of L. luteus Seeds

**Figure 2:**
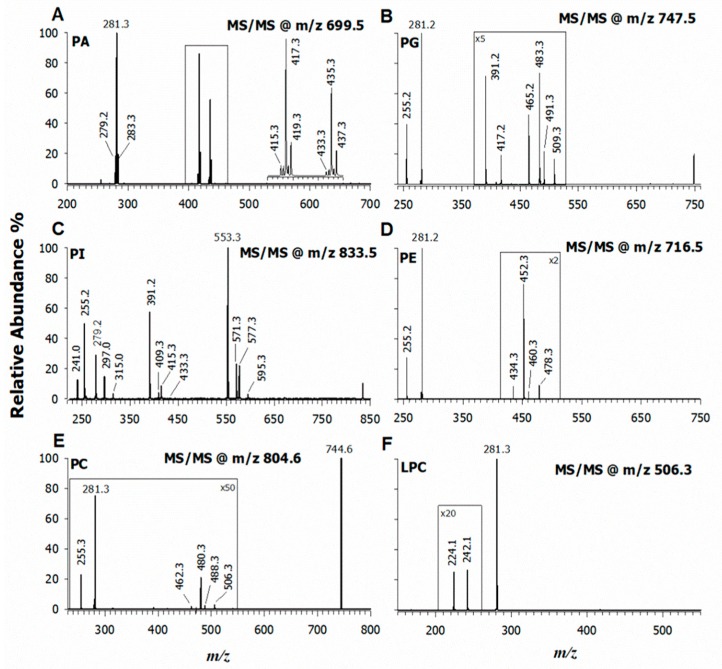
Tandem MS spectra using the low-resolution LIT of representative lipids identified in the lipid extract of *L. Luteus* seeds. Negative ion mode MS/MS spectra of PA at *m*/*z* 699.5 (**A**), PG at *m*/*z* 747.5 (**B**), PI at *m*/*z* 833.5 (**C**), PE at *m*/*z* 716.5 (**D**), PC at *m*/*z* 804.6 (**E**), and LPC at *m*/*z* 506.3 (**F**).

To establish the regiochemistry of PL, the extracted lipids of *L. luteus* seeds were investigated by collision induced dissociation (CID) tandem MS. A systematic study of PL fragmentation in both higher collisional energy dissociation (HCD) (i.e., FTMS) and CID (i.e., LITMS) energy regimes was undertaken by standard compounds, when available, or by comparison with literature data [24,25,26]. As an example, Figure 2A shows the CID-MS/MS spectrum of the precursor ion [M − H]^−^ at *m*/*z* 699.5. The accurate value at *m*/*z* 699.4970 suggested the occurrence of a phosphatidic acid, PA 36:2. In the same plot (A), three product ions can be easily recognized as carboxylate anions: 18:0 (*m*/*z* 283.3), 18:1 (*m*/*z* 281.2), and 18:2 (*m*/*z* 279.2). Conceivably, the deprotonated species at *m*/*z* 699.5 corresponds to two isobaric PA species recognized as 18:1/18:1 and 18:2-18:0 (*vide infra*). The additional product ions at *m*/*z* 435.3, 437.3, 433.3, and at *m*/*z* 417.3, 419.3, 415.3 are most likely formed upon detachment from the precursor ion of FA 18:1, 18:2, and 18:0 as neutral ketenes or fatty acids, respectively. Since the gas-phase loss of FA in the sn_2_ position of a PA is favored under the relatively low-energy regime of CID [25], the signal intensities of this carboxylate ion (i.e., [R_2_COO]^−^) and those related to its neutral loss, both as FA and as a ketene, are higher than the corresponding losses from the sn_1_ position. By applying this rule, it was possible to establish the regiochemistry of PA and to discover the presence of two isobaric PA, i.e., 18:1/18:1 and 18:0/18:2. Following this approach, all recognized PA detected in the mass spectrum of Figure S1A were characterized. The relevant data are summarized in Table 1. Additional examples of tandem MS spectra of PA are reported in Figure S2 (Supporting Information). Even if PA represent minor membrane phospholipids, they are extremely important in vegetables, since they fulfil key roles during growth, development, and against environmental stresses. PA exhibit dynamic homeostasis in plants and are involved as metabolic precursors of glycerophospholipids and as signaling messengers [27]. Due to their distinctive chemical features, PA affect the properties of membranes in regulating the intracellular distribution of proteins through their binding and plant growth modulation [28]. As a result of their characterization, it was possible to assess that the fatty acids representing the most intense signals of PA detected in *L. luteus* (Figure S1A) at *m*/*z* 683.5, 709.5, 685.5, 711.5, 707.5, and 705.5 are mainly represented by polyunsaturated fatty acyl chains, 18:2, 19:2, 18:3, and 19:3. Accordingly, recent results have proven the occurrence of straight-chain odd-number FA as minor constituents in plants [29]. In the chloroplast shell, each PA works as the precursor of a PG, which is an important constituent of the chloroplast membranes [30]. PG were detected in the chromatogram of *L. luteus* seeds at comparable content of PA (see Figure 1). The identification and structural characterization of PG were carried out as described above for PA through an initial identification by (i) retention times of the HILIC separation and (ii) accurate *m*/*z* values. The ensuing regiochemical designation was accomplished upon careful examination of each CID-MS/MS spectra. For instance, Figure 2B shows the tandem MS spectrum of the precursor ion at *m*/*z* 747.5. The online database suggested a PG (34:1) and the occurrence of fatty acids 16:0 and 18:1, at *m*/*z* 255.2 and 281.2, respectively, was promptly ensured. Furthermore, peak signals related to their losses both as carboxylic acids at *m*/*z* 491.3 and 465.3 and as ketenes at *m*/*z* 509.3 and 483.3 were detected as product ions. Since the chain detachment from the sn_2_ position is favored, the peak intensity of the [R_2_COO]^−^ ion and peak signals relevant to the losses as a fatty acid and a ketene is higher than the those of [R_1_COO]^−^ [31]. Consequently, the established regiochemistry was PG 16:0/18:1 and, in Table 1, are reported all PG identified in the seeds of yellow lupin. Another four examples of CID tandem MS spectra of PG are reported in Figure S3 (Supporting Information). The most abundant PG at *m*/*z* 745.5, 747.5, 771.5, and 773.5 are composed mainly by palmitic (16:0), oleic (18:1), and linoleic (18:2) fatty acyl chains.

The same strategy was applied to identify all the other PL species. Figure 2C shows the CID-MS/MS spectrum of the precursor ion [M − H]^−^ at *m*/*z* 833.5 recognized as PI (34:2). The product ions at *m*/*z* 255.2 and 279.2 are due to palmitic and linoleic acids as carboxylate anions, respectively. While the product ions at *m*/*z* 553.3 and 571.3 refer to the neutral loss of an FA or a ketene 18:2, respectively, the signals at *m*/*z* 577.3 and 595.3 are due to the loss of residue 16:0 as FA and ketene and their relative intensity ratio can be used to establish the occurrence of the PI 16:0/18:2. Note that all the following product ions at *m*/*z* 391.2, 409.3, 415.3, and 433.3 are formally formed upon neutral loss of dehydrated inositol (162.05 Da) from ions at *m*/*z* 553.3, 571.3, 577.3, and 595.3, respectively. Still, characteristic product ions of this PL class are produced from the polar head detected at *m*/*z* 241.0, 297.0, and 315.0, which were assigned at the following empirical formulas: [PO_4_C_6_H_10_O_4_]^−^, [PO_4_C_9_H_14_O_5_]^−^, and [PO_4_C_9_H_16_O_6_]^−^, respectively. Other examples of tandem MS spectra of PI are given in Figure S4 (Supporting Information). The most abundant PI at *m*/*z* 831.5, 833.5, 835.5, 859.5, and 861.5 were those containing palmitic (16:0), oleic (18:1), linoleic (18:2), and linolenic (18:3) acyl chains. The same rules were applied to phosphatidylethanolamines (PE), zwitterionic PL mainly distributed in extra-plastid membranes of plant cells as major non-bilayered lipids [32]. The fragmentation pathway of a precursor ion at *m*/*z* 716.5 is presented in Figure 2D (see also Figure S5, Supporting Information) and assigned to PE (16:0/18:1). Remarkably, the most abundant PE at *m*/*z* 716.5, 740.5, and 742.5 are composed of fatty acyl chains 16:0, 18:1, and 18:2 (see Table 1). 

Phosphatidylcholines (PC) are the major constituent of cell membranes in plants. Often PC are identified as lecithins, which are mainly produced from vegetable foodstuffs [33]. The term lecithin was initially used to define a sticky orange material isolated from egg yolk, while, currently, “lecithin” refers to different meanings including a mixture of PC, PE, PS, PI, other phospholipids, triglycerides, and fatty acids [34]. Currently, soybean, sunflower, and rapeseed are the main sources of commercial lecithins and *L. luteus* seeds may be suggested as a unique alternative. The identification of all the PC species was carried out in a negative ion mode exploiting the formation of formate or demethylated adducts since tandem mass spectra of [M − H]^−^ ions did not provide clear information on the acyl chains. Figure 2E illustrates the MS/MS spectrum of a PC (34:1) at *m*/*z* 804.6 as [M + HCOO]^−^. The product ion at *m*/*z* 744.6 [M − 15]^−^ was due to the radical loss of a methyl group from the choline headgroup [35,36]. Peaks detected at *m*/*z* 255.3 and 281.3 correspond to palmitic acid and oleic acid anions, respectively. Small signals detected at *m*/*z* 480.3 and 506.3 refer to the loss as ketenes of oleic [M − CH_3_ − 264]^−^ and palmitic [M − CH_3_ − 238]^−^ acids, respectively, accompanied with the relevant loss of neutral fatty acids at *m*/*z* 462.3 and 488.3. The relative intensity of carboxylate anions let us define the regiochemistry as PC 16:0/18:1 (see Table 1). Additional examples of CID tandem MS spectra of PC are reported in Figure S6 (Supporting Information).

Lastly, Figure 2F reports the CID-MS/MS spectrum of a representative LPC 18:1 at *m*/*z* 506.3. More details of the lyso-forms assignment are presented in the next paragraph. In this case, we wish to highlight that the most abundant fatty acyl chains of PC and LPC species were represented by 16:0, 18:1, and 18:2.

### 2.3. Characterization of Lysophospholipids by LC-ESI-MS/MS

**Figure 3:**
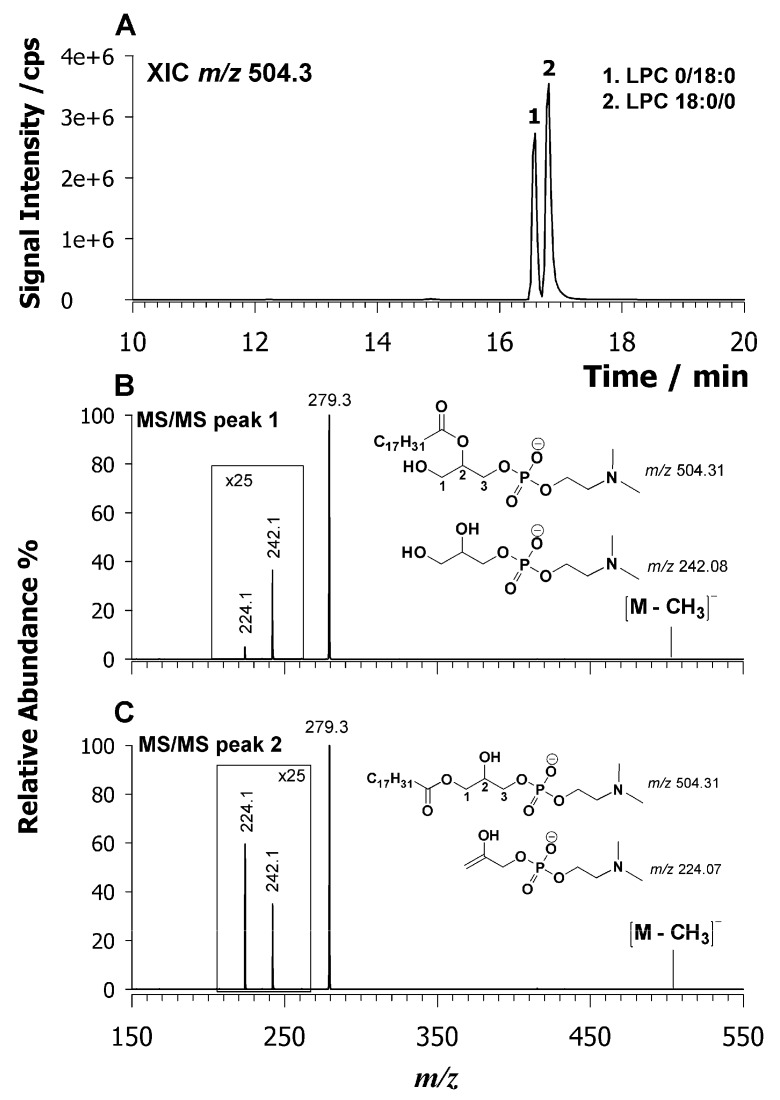
XIC chromatogram of LPC 18:2 centered at *m*/*z* 504.3 (**A**) identified in the lipid extract of *L. Luteus* seeds. Tandem MS spectra of the deprotonated molecule ([M − H]^−^) of regio-isomers LPC 0/18:2 (**B**) and 18:2/0 (**C**). The suggested chemical structures of product ions at *m*/*z* 242.1 and 224.1 are given in insets of plots (**B**,**C**).

Recently, we have demonstrated [22] the separation of lysophospholipid (LPL) regio-isomers by HILIC using a silica-based fused-core column, whereby sn_1_ isomers display higher retention times than sn_2_ ones. The chromatographic profile of Figure 1 exhibits two peaks of LPC 18:2, which suggests the occurrence of both regio-isomers even though such a circumstance needs to be confirmed by MS/MS measurements. For this reason, an extracted ion chromatogram at *m*/*z* 504.3 is reported in Figure 3A with two isobaric peaks labelled as 1 and 2. Usually, ESI-MS/MS analysis providing structural information on the regiochemistry of LPC species was performed on precursor ions as protonated adducts, [M + H]^+^ [22,37]. 

**Figure 4:**
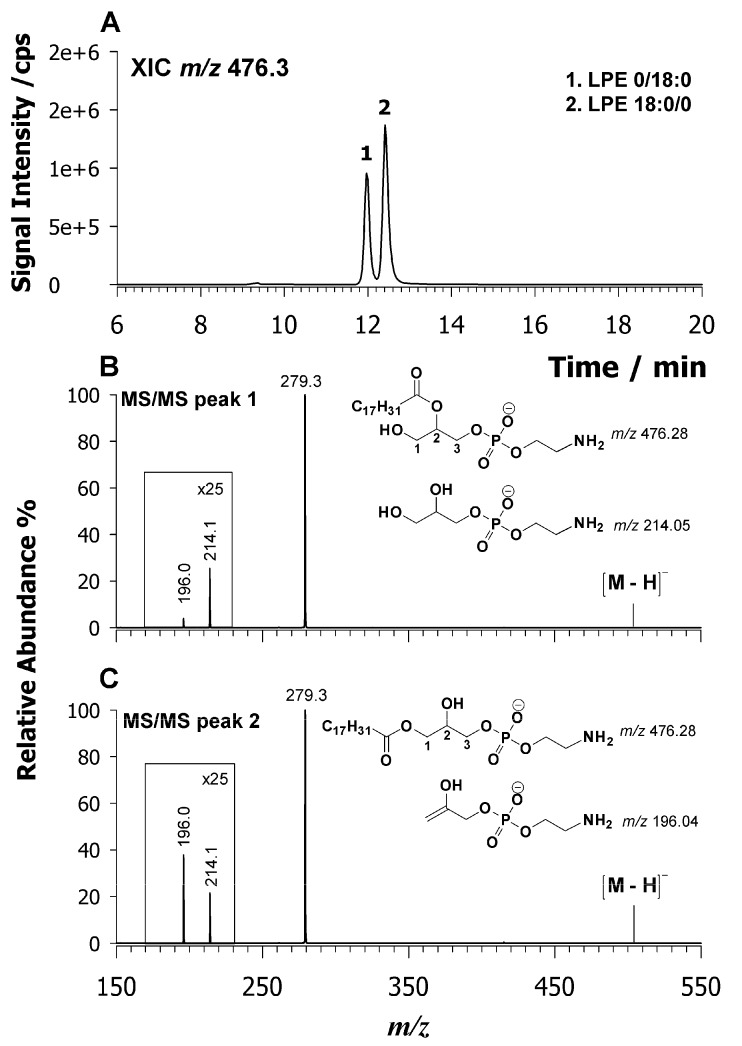
XIC chromatogram of an LPE 18:2 centered at *m*/*z* 476.3 (**A**) identified in the lipid extract of *L. Luteus* seeds. Tandem MS spectra of the deprotonated molecule ([M − H]^−^) of regio-isomers LPE 0/18:2 (**B**) and 18:2/0 (**C**). The suggested chemical structures of product ions at *m*/*z* 214.1 and 196.0 are given in insets of plots (**B**,**C**).

The diagnostic signal for the chain assignment was obtained by tandem MS and peak signal intensity of product ions [M + H − H_2_O]^+^ and a phosphocholine head group at *m*/*z* 184.07 being greater than 1 for the regio-isomer LPC sn_1_ [37]. By employing higher collision dissociation energies, a diagnostic ion at *m*/*z* 104.1 assigned to the choline ion was observed for sn_1_ LPC regio-isomers [38]. In this case, the fragmentation pattern of LPL in a negative ion mode was investigated by searching for diagnostic product ions. The CID-MS/MS spectra of both peaks 1 and 2 of LPC 18:2 are compared in plots B and C of Figure 3, respectively. In both cases, the predominant product ions occur at *m*/*z* 279.3, which is the carboxylate 18:2 anion. Two other minor signals were detected at *m*/*z* 224.1 and 242.1. These product ions are related to the polar head and are generated from the neutral loss of the acyl chain as a ketene (*m*/*z* 242.1) or as a fatty acid (*m*/*z* 224.1). The main difference between the MS/MS spectra relies on the relative intensity of these two signals 224/242 being greater than 1 only for peak 2. Usually, in HILIC separations, the sn_1_ isomers elute later than sn_2_ ones. Thus, peak 2 with signal intensity 224/242 > 1 must be assigned to the sn_1_ regio-isomer. To confirm such an assignment, a standard LPC with known sn_1_ regio-chemistry was explored. Figure S7 reports the XIC chromatogram of LPC 17:0/0 observed as a demethylated molecule at *m*/*z* 494.3 (A) along with its tandem MS spectrum (B). As expected, the intensity ratio 224/242 is greater than 1, which confirms that this ratio can be diagnostic to ascertain the LPC regio-chemistry. Turning back to LPC of Figure 3A, the chromatographic peaks 1 and 2 can be assigned as LPC 0/18:2 and LPC 18:2/0, respectively. All LPC (see Table 1) were assigned by considering the relative retention times and fragmentation behavior. Additional examples of CID tandem MS spectra of LPC are reported in Figure S8 (Supporting Information). Another example of LPL species is reported in Figure 4A, where an almost baseline separation of LPE 18:2 isomers at *m*/*z* 476.3 is shown. Two isobaric chromatographic peaks, labelled as 1 and 2, are present at retention times of 11.9 and 12.4 min, respectively. As mentioned above, the sn_2_ refers to a regio-isomer LPE eluting before the sn_1_ one [22]. Typically, LPE regio-isomers are distinguished by CID-MS/MS analysis in a positive ion mode [37] in which the fragmentation of LPE sn_1_ produces only the diagnostic product ion of the PE class as [M + H − 141]^+^ (i.e., loss of phosphoethanolamine polar head) while the fragmentation of sn_2_ regio-isomer generates an intense product ion due to water loss. Thus, the relative intensity of both major product ions [M + H − 141]^+^ and [M + H − H_2_O]^+^ allowed us to distinguish between the LPE regio-isomers [37]. In our case, the deprotonated molecule, [M − H], was investigated by CID-MS/MS and results are reported in Figure 4 and plots B and C. In both spectra, the main product ion detected is due to 18:2 carboxylate anion at *m*/*z* 279.2. Two additional minor signals were detected at *m*/*z* 196.1 and 214.0. These product ions are related to the PE head and explained as the neutral loss of the acyl chain as ketene (*m*/*z* 214.0) or fatty acid (*m*/*z* 196.1). The main difference between these spectra is given by the relative intensity of these product ions, 196.1/214.0, which is higher than 1 for peak 2 that identifies the sn_1_ regio-isomer on the base of HILIC retention time. For a full confirmation of the tandem MS outcomes, LPE with known sn_1_ regio-chemistry was examined. Figure S9 (Supporting Information) reports the XIC chromatogram of the LPE 13:0/0 observed as a deprotonated molecule at *m*/*z* 410.2 (A) along with the CID-MS/MS spectrum (B). Even if the main product ion at *m*/*z* 213.2 due to carboxylate C13:0 interferes with a peak at *m*/*z* 214.0, it is still possible to realize as the intensity ratio 196.1/214.0 is greater than 1, which confirms the sn_1_ regiochemistry. Therefore, the LPE at *m*/*z* 476.3 in the lipid extract of *L. luteus* is 0/18:2 for peak 1 and 18:2/0 for peak 2 (Figure 4A).

The CID-MS/MS of LPC and LPE by HCD tandem FTMS in negative ion mode were carried out as well (data not shown). The results confirmed that intensity ratios 224.1/242.1 (for LPC) and 196.1/214.0 (for LPE) are greater than 1 in the case of sn_1_ regio-isomers. By such an approach, the identification of previously uncharacterized 16 LPC and 14 LPE in *L. luteus* seed extracts was possible. 

To estimate the average distribution of PL species in *L. luteus* seeds, five different samples were analyzed in triplicate. For each sample, the XIC trace related to each PL class was generated. The area of each peak was integrated and the relative abundance (%) on all detected species was computed. The relative distribution of PL classes was the following: PC (41 ± 6%), LPC (30 ± 11%), PE (13 ± 4%), PG (5.1 ± 1.7%), PA (4.9 ± 1.8%), PI (4.7 ± 1.1%), and LPE (1.2 ± 0.5%). A relatively high content of LPC was observed in contrast to the mean values of white lupin seeds reported by Hamama and Bhardwaj [13] using TLC. The generation of LPL from glycerophospholipids is catalysed by phospholipases A (PLA) normally occurring in plant tissues. These enzymes are comprised in the superfamily of phospholipase A1 (PLA_1_) and phospholipase A2 (PLA_2_), which catalyse the phospholipids’ hydrolysis at either the sn_1_ or sn_2_ position, respectively [39]. Despite PLA_2_ being more active with prevalent formation of sn_1_ lyso-forms of LPC, LPE, and LPI, the present data demonstrated the occurrence of both LPL regio-isomers with only a slight prevalence of sn_1_ LPL. Normally, LPL are present in membranes only in trace amounts but their level increases during adaptation to freezing [40], in response to wounding [41], during cell expansion [42], or as a result of pathogen infection [43]. Since *Lupinus luteus* L. is mostly cultivated in sandy and acidic soils, and wild populations are much less common than *L. albus* [44], yellow lupin is a more resistant plant to harsh environmental conditions. Nonetheless, more data are needed on the biochemistry and physiological functions of lupin PLA to understand the LPL signalling cascades [45]. Until today, approximately 20 plant PLA_2_ are reported [46]. Thus, the study of these enzymes and their activity in lupin seeds will allow us to better clarify their involvement.

### 2.4. RPLC-ESI-MS/MS of Linked Fatty Acyl Chains upon Chemical Hydrolysis

**Figure 5:**
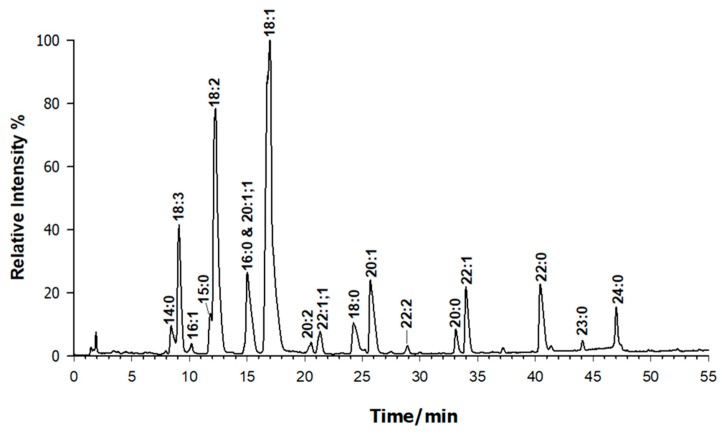
Representative RPLC-ESI/MS in total ion current (TIC) of a chemically hydrolyzed lipidic extract of *L. Luteus* seeds (see Experimental section) with the ensuing formation of fatty acids as labelled in the figure. Peaks labelled as 20:1;1 and 22:1;1 are those containing a double bond and a hydroxyl moiety.

The linked fatty acyl chains of PL in the extracted sample were hydrolysed in acidic solution and analysed by RPLC-ESI-FTMS in a negative polarity mode, detecting each FA as a deprotonated molecule, [M − H]^−^. As an example, Figure 5 reports a typical TIC profile of a sample solution treated with HCl 0.5 M at 100 °C for 45 min. The accurate *m*/*z* ratios collected over the entire chromatogram were used as input data for the online lipid search to assign the FA empirical formula. The tolerance was purposely set as the lowest possible ±0.001 *m*/*z* unit since the fatty acids will be identified from the accurate *m*/*z* values. As a result, all *m*/*z* ratios detected in the first 55 min of the TIC trace were compatible with FA of variable chemical compositions. The most intensely detected peaks were labelled by adopting the conventional C:D nomenclature in which C indicates the number of carbon atoms and D indicates the number of C=C bonds for each FA. The peak’s identification was confirmed by comparing their retention times with those of a standard FA solution, as previously reported [20]. As anticipated, fatty acids are separated in RPLC both by chain length and by the degree of unsaturation. Therefore, the presence of a double bond reduces the effective chain length by nearly two carbon units as testified by FA 18:1 that elutes after FA 16:0. Additional double bonds have smaller effects on retention. For instance, the FA 18:3 elutes before FA 15:0 or FA 16:1. According to previous data focused on FA of lupin seeds [13,47], the occurrence of FA from 14 to 24 C atoms was confirmed. The presence of minor components of FA with an odd number of carbon atoms as 15:0 and 17:0 on their chains is reported. Their existence could be related to different sources including bacteria since odd-numbered FA, often C15:0, C17:0 (i-, ai- and n-), and C19 are mostly due to contamination of microorganisms. This is not surprising because one gram of soil contains roughly 40 million of bacteria, and both water and air are symbiotic to multicellular organisms as plants [29]. Moreover, hydroxylated FA acids are observed in the chromatogram, which are not present in PL. Their occurrence may be due to the concurrent hydrolysis of other lipid species including ceramides.

To estimate the relative content of FA, the eXtracted Ion Current (XIC) chromatograms were systematically examined for each detected *m*/*z* ratio and the peak area was related to the total one. As can be seen from Table 2, where all FA data are reported, oleic acid (18:1) is present in significant amounts in lupin seeds (38.2 ± 4.5%), which is followed by the essential linoleic (18:2), linolenic (18:3), palmitic (16:0) acids, followed by gondoic (20:1), behenic (22:0), and erucic (22:1) acids with relevant amounts of even longer FA as lignoceric acid (24:0). The resulting PUFA + MUFA/SFA ratio was found on average to be 5.0 ± 0.5 for all the analysed samples, which confirms the nutritional value of yellow lupins.

### 2.5. Lupin Seeds as an Alternative Source of PL

Taken the current lack of data on the content of bioactive lipids in yellow lupin seeds, the present study is a preliminary effort in this direction. Soybean is another seed belonging to the Fabaceae family that is the greatest source of PL providing about 200,000 tons per year, and almost 90% of the total market. As mentioned earlier, an alternative PL source for dietetic purposes, infant formulas, and parenteral nutrition, is given by egg lecithins. Yet, the production is limited to 300 tons per year [48]. Generally, soybean lecithin contains PC (20–22%) being PC 18:2/18:2 the main component, PE (16%–22%), PI (13%–16%), PA (5%–10%), and small amounts of LPC (around 3%) while egg lecithins contain a high amount of PC (75–85%), which is followed by PE (12%–17%) and lower amounts of sphingomyelins (SM), LPE, and LPC [49]. These PL extracted from food products are defined as “dietary PL” and are often sold as food supplements. As anticipated above and considering that the costs of PL isolated from natural sources is always lower than that obtained by synthetic or semi-synthetic methods, it can be deduced that lupin may represent a valuable source of lecithins with intermediate features between soy and egg.

## 3. Materials and Methods

### 3.1. Chemicals

Water, acetonitrile, methanol, chloroform, formic acid, ammonium formate, and ammonium acetate were obtained from Sigma-Aldrich (Milan, Italy). *Lupinus luteus* seeds of various origins were purchased from local or biological supermarkets. Standard lipids phosphatidylglycerol (PG) 38:4, phosphatidylethanolamine (PE) 28:0, phosphatidylcholine (PC) 36:1, lyso-PC 17:0, and lyso-PE 13:0 were obtained from Spectra 2000 SRL (Rome, Italy). All solvents used were LC–MS grade except for CHCl_3_ and methyl-tert-butyl-ether (MTBE) (HPLC grade). A calibrating solution containing caffeine, methionine-arginine-phenylalanine-alanine peptide, and Ultramark as well as a mixture of fluorinated phosphazines were purchased from Thermo Scientific (Waltham, MA, United States). 

### 3.2. Sample Preparation

#### 3.2.1. Lipid Extraction

Following the Bligh &Dyer (BD) protocol [50], 3 mL of methanol/chloroform (2:1, *v*/*v*) were added to 1.5 g of ground lupin seeds that were diluted with 800 µL of water. The seeds were washed at least four times to remove salts from the storage saline solution. Then, 1 mL of chloroform was added, and the mixture was vortexed for 30 s. Lastly, 1 mL of water was added, and the solution was shaken before being centrifuged for 10 min at 3000× *g*. The lower phase containing lipids was dried under nitrogen flow and subjected to solid phase extraction (SPE) purification for PL analysis or chemical hydrolysis for analysing fatty acids.

#### 3.2.2. SPE Purification

To reduce the high salt concentration from preservation liquid, a micro solid phase extraction (μSPE) was carried out on the lipid extract [51]. Specifically, 40 mg of silica were weighted and packed in a tip. The micro column was conditioned with 500 μL of MTBE/CHCl_3_/CH_3_COOH solution (98/2/0.05, *v*/*v*/*v*). The dried sample was dissolved in 200 μL of the same solution and loaded. The column was then washed first with 500 μL of the same solution and then with 250 μL of MTBE. The elution was carried out with 700 μL of MTBE/CHCl_3_/CH_3_OH solution (50/20/30, *v*/*v*/*v*). The eluate was dried under a flux of nitrogen and then dissolved back in 50 μL of propanol/CHCl_3_/CH_3_OH solution (90/5/5).

#### 3.2.3. Chemical Hydrolysis of Linked Fatty Acyl Chains

The dried product resulting from BD extraction was taken up in 1 mL of HCl 0.5 M in ACN: H_2_O (9:1, *v*:*v*) and left at 100 °C for 45 min. Then 1 mL of CHCl_3_ and 1 mL of H_2_O were added, and the mixture was centrifuged for 15 min at 3000× *g*. The organic phase was collected. A total of 1 mL of H_2_O was added, vigorously mixed, and then centrifuged again for 15 min at 3000× *g*. The organic phase was recovered, dried under nitrogen, and dissolved in 1 mL of CHCl_3_:MeOH (1:1) for successive RPLC analysis.

### 3.3. LC-ESI-MS Instrumentation and Operating Conditions

HILIC-ESI-FTMS measurements were performed using an LC-MS apparatus consisting of an UHPLC system Ultimate 3000 and a hybrid Q-Exactive mass spectrometer (Thermo Scientific, Waltham, MA, USA), equipped with a heated electrospray ionization (HESI) source and a higher collisional energy dissociation (HCD) cell for tandem MS analyses. Chromatographic separations were run at ambient temperature (25 ± 1 °C) on a narrow-bore column (150 × 2.1 mm ID, 2.7 μm particle size) equipped with a security guard cartridge (5 × 2.1 mm ID) both Ascentis Express HILIC (Supelco, Bellefonte, PA, USA) using a flow rate of 0.3 mL min^−1^. A volume of 5 μL of the lupin lipid extract was injected into the column using a Rapid Separation (RS) Autosampler (Thermo Scientific). The adjusted binary elution program, based on water, and 2.5 mmol L^−1^ ammonium formate (solvent A) and acetonitrile (solvent B), both containing 0.1% (*v*/*v*) of formic acid, was adopted [52]: 0–5 min, linear gradient from 97 to 88% solvent B, 5–10 min, isocratic at 88% solvent B, 10–11 min, linear gradient from 88 to 81% solvent B, 11–20 min, linear gradient from 81% to 70% solvent B, 20–22 min, linear gradient from 70 to 50% solvent B, 22–28 isocratic at 50% solvent B; 28–30 min, return to the initial composition, followed by a 5-min equilibration time. 

Fatty acids were separated by reversed-phase liquid chromatography (RPLC) using a Supelco (Bellefonte, PA, USA) Ascentis Express C18 column (150 × 2.1 mm ID, 2.7-µm particle size). A binary gradient elution, based on water (solvent A) and methanol (solvent B), both containing 2.5 mM of ammonium acetate, was optimized, on a multi-FA standard mixture [20]. The following program was finally adopted: 0–50 min, linear from 80% to 100% B, 50–60 min, isocratic at 100% B, 60–65 min, return to the initial composition, and followed by a 20-min equilibration time. The flow rate was 0.2 mL/min. The column effluent was transferred into the Q-Exactive spectrometer through the HESI source. The main ESI and ion optic parameters were the following: sheath gas flow rate, 35 (arbitrary units, a.u.), auxiliary gas flow rate, 15 a.u., spray voltage, 3.5 kV (positive) and −2.5 kV (negative), capillary temperature, 320 °C, S-lens radio frequency level, 100 a.u. Negative and positive MS full-scan spectra were acquired in the *m*/*z* range 130–2000, after setting a mass resolving power of 140,000 (at *m*/*z* 200). The instrument was daily calibrated and mass accuracies ranged between 0.10 and 0.15 ppm in positive polarity and between 0.40 and 0.45 ppm in a negative polarity. Peak area referred to specific FA or PL species were obtained from eXtracted Ion Current (XIC) chromatographic traces resulting, respectively, from RPC or HILIC-ESI-FTMS analysis and were normalized on each total area. Besides accurate masses, LC-MS runs using targeted-MS^2^ acquisitions that were performed to confirm the phospholipid species. In this modality, the *m*/*z* values of the selected precursor ions were introduced into an inclusion list, each with a tolerance of 10 ppm. MS/MS measurements were run using a 1 *m*/*z* unit wide window, a resolving power of 70,000 (at *m*/*z* 200), a fill time of 100 ms, and Automated Gain Control (AGC) of 2 × 10^5^. Further HILIC-MS measurements were performed in parallel using a medium resolving power and mass accuracy apparatus, including the UHPLC system Ultimate 3000 coupled to a Velos Pro mass spectrometer (Thermo Scientific) equipped with a linear ion trap analyzer and a HESI interface. This instrument was used since the double-stage linear ion trap mass analyzer working in a low-energy collisional induced dissociation (CID) regime was complementary to HCD to confirm some doubtful attributions. Only the S-lens radio frequency level, lowered to 60 (arbitrary units), was modified among HESI and ion optic parameters when using the Velos Pro spectrometer. Collision energy varied according to the ion of interest, from 35% to 45% (in this case, a 400% value corresponds to a 100 V excitation voltage) using a 1 *m*/*z* unit wide isolation window centered on the *m*/*z* ratio. 

### 3.4. Preliminary Identification of PL by an Online Lipid Calculator

A preliminary identification of lipids (i.e., PL, LPL, and FA) extracted from *L. luteus* seeds was performed using the Online Lipid Calculator, available freely at the following address: www.mslipidomics.info/lipid-calc/. Given a certain *m*/*z* value and tolerance, the software retrieves the possible candidate structures among several lipid classes. The software calculates different positively or negatively charged adducts relevant to the *m*/*z* values in input. In the present study, the input values correspond to accurate *m*/*z* ratios retrieved from MS spectra obtained with the Q-Exactive spectrometer.

## 4. Conclusions

The characterization of phospholipids and fatty acyl chains in *L. luteus* seeds by LC-ESI-MS was accomplished and more than 200 main phospholipids were regiochemically identified. As far as the FA composition, it differs among PL classes. Whereas 18:1 and 18:2 acyl chains were present in the most abundant molecular species of PI, PG, PE, PC, and LPC, polyunsaturated acyl chains 18:2, 18:3, 19:2, and 19:3 were the most abundant in PA. Regio-isomers of LPC and LPE were identified, which proposed a featured fragmentation pattern in - negative ion mode and the higher content of lyso- sn_1_ forms emphasized greater activity of PLA_2_ in plants. With the aid of a chemically hydrolyzed mixture of a lipid extract, all minor and major fatty acids were released, and their LC-ESI-MS examination agreed with the fact that 18:1 and 18:2, most likely oleic and linoleic acids, were the most abundant fatty acyl chains. A quantitative analysis of PL and LPL species and a comparison among other lupin species such as *L. albus* and *L. angustifolius* are planned soon.

## Figures and Tables

**Table 1:** Summary of phospholipids identified in *L. Luteus* seeds by HILIC-ESI-MS/MS in a negative ion mode. Accurate masses, lipid species, adduct observed, and sum formula are reported.

Lipid Species	Accurate *m*/*z* Value	Adduct	Empirical Formula (M)	Lipid Species
1	671.4672	[M − H]^−^	C_37_H_68_O_8_P	PA (16:0/18:2)
2	673.4825	[M − H]^−^	C_37_H_70_O_8_P	PA (16:0/18:1)
3	683.4673	[M − H]^−^	C_38_H_68_O_8_P	PA (17:0/18:3) PA (19:3/16:0)
4	685.4827	[M − H]^−^	C_38_H_70_O_8_P	PA (19:2/16:0) PA (17:0/18:2)
5	687.4986	[M − H]^−^	C_38_H_72_O_8_P	PA (17:0/18:1) PA (19:1/16:0)
6	695.4658	[M − H]^−^	C_39_H_68_O_8_P	PA (18:2/18:2) PA (18:1/18:3) PA (18:3/18:1)
7	697.4816	[M − H^−^	C_39_H_70_O_8_P	PA (18:1/18:2) PA (18:2/18:1) PA (18:0/18:3) PA (20:3/16:0)
8	699.4978	[M − H]^−^	C_39_H_72_O_8_P	PA (18:0/18:2) PA (18:1/18:1)
9	701.5136	[M − H]^−^	C_39_H_74_O_8_P	PA (18:1/18:0) PA (16:0/20:1)
10	705.4511	[M − H]^−^	C_40_H_66_O_8_P	PA (19:3/18:3)
11	707.4674	[M − H]^−^	C_40_H_68_O_8_P	PA (19:2/18:3)
12	709.4823	[M − H]^−^	C_40_H_70_O_8_P	PA (19:1/18:3) PA (19:2/18:2) PA (19:3/18:1)
13	711.4983	[M − H]^−^	C_40_H_72_O_8_P	PA (19:1/18:2) PA (19:2/18:1) PA (19:0/18:3) PA (21:3/16:0)
14	713.5132	[M − H]^−^	C_40_H_74_O_8_P	PA (19:1/18:1) PA (19:0/18:2) PA (19:2/18:0) PA (21:2/16:0) PA (20:2/17:0)
15	723.4969	[M − H]^−^	C_41_H_72_O_8_P	PA (20:2/18:2) PA (20:3/18:1) PA (20:1/18:3)
16	725.5129	[M − H]^−^	C_41_H_74_O_8_P	PA (20:1/18:2) PA (20:2/18:1)
17	727.5290	[M − H]^−^	C_41_H_76_O_8_P	PA (20:1/18:1) PA (18:2/20:0)
18	775.5318	[M − H]^−^	C_45_H_76_O_8_P	PA (24:5/18:1) PA (24:6/18:0)
19	665.4416	[M − H]^−^	C_34_H_66_O_10_P	PG (14:0/14:0) PG (12:0/16:0)
20	719.4879	[M − H]^−^	C_38_H_72_O_10_P	PG (16:1/16:0) PG (18:1/14:0)
21	721.5038	[M − H]^−^	C_38_H_74_O_10_P	PG (16:0/16:0)
22	731.4884	[M − H]^−^	C_39_H_72_O_10_P	PG (18:2/15:0) PG (18:1/15:1) PG (16:0/17:2) PG (16:1/17:1)
23	733.5033	[M − H]^−^	C_39_H_74_O_10_P	PG (15:0/18:1) PG (17:1-16:0)
24	741.4721	[M − H]^−^	C_40_H_70_O_10_P	PG (16:2/18:2) PG (16:1/18:3)
25	743.4891	[M − H]^−^	C_40_H_72_O_10_P	PG (18:3/16:0) PG (18:2/16:1)
26	745.5048	[M − H]^−^	C_40_H_74_O_10_P	PG (16:0/18:2)
27	747.5192	[M − H]^−^	C_40_H_76_O_10_P	PG (16:0/18:1)
28	757.5022	[M − H]^−^	C_41_H_74_O_10_P	PG (17:2/18:1) PG (17:1/18:2)
29	759.5171	[M − H]^−^	C_41_H_76_O_10_P	PG (18:2/17:0) PG (18:1/17:1) PG (19:2/16:0)
30	761.5332	[M − H]^−^	C_41_H_78_O_10_P	PG (18:1/17:0) PG (19:1/16:0)
31	767.4868	[M − H]^−^	C_42_H_72_O_10_P	PG (18:3/18:2)
32	769.5028	[M − H]^−^	C_42_H_74_O_10_P	PG (18:2/18:2) PG (18:1/18:3)
33	771.5188	[M − H]^−^	C_42_H_76_O_10_P	PG (18:2/18:1)
34	773.5341	[M − H]^−^	C_42_H_78_O_10_P	PG (18:1/18:1) PG (16:0/20:2) PG (20:2/16:0)
35	775.5488	[M − H]^−^	C_42_H_80_O_10_P	PG (18:0/18:1) PG (16:0/20:1)
36	777.5655	[M − H]^−^	C_42_H_82_O_10_P	PG (18:0/18:0) PG (20:0/16:0)
37	831.5037	[M − H]^−^	C_43_H_76_O_13_P	PI (16:0/18:3)
38	833.5196	[M − H]^−^	C_43_H_78_O_13_P	PI (16:0/18:2)
39	835.5352	[M − H]^−^	C_43_H_80_O_13_P	PI (16:0/18:1)
40	855.5022	[M − H]^−^	C_45_H_76_O_13_P	PI (18:3/18:2)
41	857.5183	[M − H]^−^	C_45_H_78_O_13_P	PI (18:2/18:2) PI (18:1/18:3)
42	859.5342	[M − H]^−^	C_45_H_80_O_13_P	PI (18:1/18:2)
43	861.5497	[M − H]^−^	C_45_H_82_O_13_P	PI (18:1/18:1) PI (18:0/18:2)
44	863.5629	[M − H]^−^	C_45_H_84_O_13_P	PI (18:0/18:1)
45	634.4502	[M − H]^−^	C_33_H_65_NO_8_P	PE (12:0/16:0) PE (10:0/18:0)
46	674.4722	[M − H]^−^	C_36_H_69_NO_8_P	PE (13:0/18:1) PE (15:1/16:0)
47	686.4771	[M − H]^−^	C_37_H_69_NO_8_P	PE (14:0/18:2) PE (16:1/16:1)
48	688.4927	[M − H]^−^	C_37_H_71_NO_8_P	PE (14:0/18:1) PE (16:0/16:1)
49	702.5085	[M − H]^−^	C_38_H_73_NO_8_P	PE (15:0/18:1) PE (16:0/17:1)
50	712.4938	[M − H]^−^	C_39_H_71_NO_8_P	PE (16:0/18:3)
51	714.5097	[M − H]^−^	C_39_H_73_NO_8_P	PE (16:0/18:2) PE (16:1-18:1)
52	716.5244	[M − H]^−^	C_39_H_75_NO_8_P	PE (16:0/18:1)
53	718.5385	[M − H]^−^	C_39_H_77_NO_8_P	PE (16:0/18:0)
54	738.5097	[M − H]^−^	C_41_H_73_NO_8_P	PE (18:2/18:2) PE (18:3/18:1)
55	740.5246	[M − H]^−^	C_41_H_75_NO_8_P	PE (18:2/18:1)
56	742.5395	[M − H]^−^	C_41_H_77_NO_8_P	PE (18:1/18:1)
57	744.5521	[M − H]^−^	C_41_H_79_NO_8_P	PE (18:0/18:1)
58	754.5402	[M − H]^−^	C_42_H_77_NO_8_P	PE (19:2/18:1) PE (19:1/18:2) PE (17:3/20:0)
59	766.5387	[M − H]^−^	C_43_H_77_NO_8_P	PE (18:0/20:4) PE (18:1/20:3)
60	768.5540	[M − H]^−^	C_43_H_79_NO_8_P	PE (20:1/18:2) PE (20:2/18:1) PE (18:0/20:3) PE (20:0/18:3)
61	770.5699	[M − H]^−^	C_43_H_81_NO_8_P	PE (20:1/18:1) PE (20:0/18:2)
62	776.5255	[M − H]^−^	C_44_H_75_NO_8_P	PE (21:5/18:1) PE (23:6/16:0)
63	778.5388	[M − H]^−^	C_44_H_77_NO_8_P	PE (21:5/18:0) PE (21:4/18:1)
64	794.5709	[M − H]^−^	C_45_H_81_NO_8_P	PE (18:1/22:3) PE (18:0/22:4) PE (18:2/22:2) PE (18:3/22:1)
65	796.5862	[M − H]^−^	C_45_H_83_NO_8_P	PE (22:1/18:2) PE (22:0/18:3) PE (22:2/18:1)
66	452.2793	[M − H]^−^	C_21_H_43_NO_7_P	LPE (16:0/0:0)
67	452.2793	[M − H]^−^	C_21_H_43_NO_7_P	LPE (0:0/16:0)
68	474.2639	[M − H]^−^	C_23_H_41_NO_7_P	LPE (18:3/0:0)
69	474.2639	[M − H]^−^	C_23_H_41_NO_7_P	LPE (0:0/18:3)
70	476.2792	[M − H]^−^	C_23_H_43_NO_7_P	LPE (18:2/0:0)
71	476.2792	[M − H]^−^	C_23_H_43_NO_7_P	LPE (0:0/18:2)
72	478.2947	[M − H]^−^	C_23_H_45_NO_7_P	LPE (18:1/0:0)
73	478.2947	[M − H]^−^	C_23_H_45_NO_7_P	LPE (0:0/18:1)
74	480.3055	[M − H]^−^	C_23_H_47_NO_7_P	LPE (18:0/0:0)
75	480.3055	[M − H]^−^	C_23_H_47_NO_7_P	LPE (0:0/18:0)
76	506.3262	[M − H]^−^	C_25_H_49_NO_7_P	LPE (20:1/0:0)
77	506.3262	[M − H]^−^	C_25_H_49_NO_7_P	LPE (0:0/20:1)
78	536.3734	[M − H]^−^	C_27_H_55_NO_7_P	LPE (0:0/22:0)
79	712.4932	[M − CH_3_]^−^	C_39_H_71_NO_8_P	PC (14:2/18:1) PC (14:1/18:2) PC (14:0/18:3)
80	714.5087	[M − CH_3_]^−^	C_39_H_73_NO_8_P	PC (14:0/18:2) PC (16:1/16:1)
81	716.5245	[M − CH_3_]^−^	C_39_H_75_NO_8_P	PC (18:1/14:0) PC (16:0/16:1)
82	718.5407	[M − CH_3_]^−^	C_39_H_77_NO_8_P	PC (16:0/16:0)
83	740.5252	[M − CH_3_]^−^	C_41_H_75_NO_8_P	PC (16:0/18:3) PC (16:1/18:2)
84	742.5414	[M − CH_3_]^−^	C_41_H_77_NO_8_P	PC (16:0/18:2) PC (18:1/16:1)
85	744.5568	[M − CH_3_]^−^	C_41_H_79_NO_8_P	PC (16:0/18:1)
86	766.5404	[M − CH_3_]^−^	C_43_H_77_NO_8_P	PC (18:2/18:2) PC (18:3-18:1)
87	768.5563	[M − CH_3_]^−^	C_43_H_79_NO_8_P	PC (18:2/18:1)
88	770.5711	[M − CH_3_]^−^	C_43_H_81_NO_86_P	PC (18:1/18:1)
89	778.5607	[M + HCOO]^−^	C_41_H_81_NO_10_P	PC (16:0/16:0)
90	790.5609	[M + HCOO]^−^	C_42_H_81_NO_10_P	PC (15:0/18:1) PC (17:1/16:0)
91	792.5766	[M + HCOO]^−^	C_42_H_83_NO_10_P	PC (17:0/16:0)
92	794.5707	[M − CH_3_]^−^	C_45_H_81_NO_8_P	PC (18:3/20:1) PC (18:2/20:2) PC (18:1/20:3)
93	796.5860	[M − CH_3_]^−^	C_45_H_83_NO_8_P	PC (20:1/18:2) PC (20:2/18:1)
94	802.5621	[M + HCOO]^−^	C_43_H_81_NO_10_P	PC (16:0/18:2) PC (16:1/18:1)
95	804.5772	[M + HCOO]^−^	C_43_H_83_NO_10_P	PC (16:0/18:1)
96	806.5891	[M + HCOO]^−^	C_43_H_85_NO_10_P	PC (16:0/18:0)
97	826.5620	[M + HCOO]^−^	C_45_H_81_NO_10_P	PC (18:2/18:2) PC (18:1/18:3)
98	828.5774	[M + HCOO]^−^	C_45_H_83_NO_10_P	PC (18:2/18:1)
99	830.5918	[M + HCOO]^−^	C_45_H_85_NO_10_P	PC (18:1/18:1)
100	832.6054	[M + HCOO]^−^	C_45_H_87_NO_10_P	PC (18:0/18:1)
101	856.6060	[M + HCOO]^−^	C_47_H_87_NO_10_P	PC (18:2/20:1) PC (18:1/20:2)
102	858.6216	[M + HCOO]^−^	C_47_H_89_NO_10_P	PC (20:1/18:1) PC (20:0/18:2) PC (19:1/19:1)
103	884.6380	[M + HCOO]^−^	C_49_H_91_NO_10_P	PC (18:2/22:1) PC (18:3/22:0) PC (18:1/22:2)
104	886.6539	[M + HCOO]^−^	C_49_H_93_NO_10_P	PC (18:2/22:0) PC (18:1/22:1)
105	888.6692	[M + HCOO]^−^	C_49_H_95_NO_10_P	PC (18:1/22:0)
106	910.6549	[M + HCOO]^−^	C_51_H_93_NO_10_P	PC (24:1/18:3) PC (24:2/18:2) PC (24:3/18:1)
107	914.6864	[M + HCOO]^−^	C_51_H_97_NO_10_P	PC (24:0/18:2) PC (24:1/18:1)
108	916.7018	[M + HCOO]^−^	C_51_H_99_NO_10_P	PC (18:1/24:0)
109	480.3104	[M − CH_3_]^−^	C_23_H_47_NO_7_P	LPC (16:0/0:0)
110	480.3104	[M − CH_3_]^−^	C_23_H_47_NO_7_P	LPC (0:0/16:0)
111	504.3109	[M − CH_3_]^−^	C_25_H_47_NO_7_P	LPC (18:2/0:0)
112	504.3109	[M − CH_3_]^−^	C_25_H_47_NO_7_P	LPC (0:0/18.2)
113	506.3253	[M − CH_3_]^−^	C_25_H_49_NO_7_P	LPC (18:1/0:0)
114	506.3253	[M − CH_3_]^−^	C_25_H_49_NO_7_P	LPC (0:0/18:1)
115	534.3567	[M − CH_3_]^−^	C_27_H_53_NO_7_P	LPC (20:1/0:0)
116	534.3567	[M − CH_3_]^−^	C_27_H_53_NO_7_P	LPC (0:0/20:1)
117	536.3725	[M − CH_3_]^−^	C_27_H_55_NO_7_P	LPC (20:0/0:0)
118	536.3725	[M − CH_3_]^−^	C_27_H_55_NO_7_P	LPC (0:0/20:0)
119	540.3312	[M + HCOO]^−^	C_25_H_51_NO_9_P	LPC (16:0/0:0)
120	540.3312	[M + HCOO]^−^	C_25_H_51_NO_9_P	LPC (0:0/16:0)
121	564.3311	[M + HCOO]^−^	C_27_H_51_NO_9_P	LPC (18:2/0:0)
122	564.3311	[M + HCOO]^−^	C_27_H_51_NO_9_P	LPC (0:0/18:2)
123	566.3465	[M + HCOO]^−^	C_27_H_53_NO_9_P	LPC (18:1/0:0)
124	566.3465	[M + HCOO]^−^	C_27_H_53_NO_9_P	LPC (0:0/18:1)

**Table 2:** Overview of linked fatty acyl chains of PL and LPL identified upon chemical hydrolysis by RPLC-ESI-MS/MS in a negative ion mode. The most abundant species are highlighted in bold.

Accurate *m*/*z*	Theoretical *m*/*z*	Accuracy (ppm)	Time (min)	FA Composition	Empirical Formula (M)	Relative Abundance (%)
199.1706	199.1704	+1.0	4.55	12:0	C_12_H_23_O_2_	0.02 ± 0.01
227.2018	227.2017	+0.4	8.40	14:0	C_14_H_27_O_2_	0.15 ± 0.01
241.2177	241.2173	+1.7	11.69	15:0	C_15_H_29_O_2_	0.11 ± 0.02
253.2179	253.2173	+2.4	10.16	16:1	C_16_H_29_O_2_	0.74 ± 0.03
**255.2332**	**255.2330**	**+0.8**	**15.08**	**16:0**	**C_16_H_31_O_2_**	**6.84 ± 0.08**
267.2339	267.2330	+3.4	13.72	17:1	C_17_H_31_O_2_	0.09 ± 0.02
269.2493	269.2486	+2.6	20.15	17:0	C_17_H_33_O_2_	0.08 ± 0.01
271.2284	271.2279	+1.8	7.96	16:0;1	C_16_H_31_O_3_	0.39 ± 0.13
**277.2173**	**277.2173**	**0.0**	**9.13**	**18:3**	**C_18_H_29_O_2_**	**9.36 ± 0.19**
**279.2330**	**279.2330**	**0.0**	**12.30**	**18:2**	**C_18_H_31_O_2_**	**21.4 ± 1.6**
**281.2487**	**281.2486**	**+0.4**	**16.96**	**18:1**	**C_18_H_33_O_2_**	**38.2 ± 4.5**
**283.2642**	**283.2643**	**−0.4**	**24.22**	**18:0**	**C_18_H_35_O_2_**	**1.23 ± 0.03**
305.2491	305.2486	+1.6	15.95	20:3	C_20_H_33_O_2_	0.11 ± 0.01
307.2646	307.2643	+1.0	20.58	20:2	C_20_H_35_O_2_	0.67 ± 0.12
**309.2801**	**309.2799**	**+0.6**	**25.67**	**20:1**	**C_20_H_37_O_2_**	**6.03 ± 0.19**
**311.2957**	**311.2956**	**+0.3**	**33.10**	**20:0**	**C_20_H_39_O_2_**	**1.03 ± 0.16**
325.2749	325.2748	+0.3	14.99	20:1;1	C_20_H_37_O_3_	0.80 ± 0.20
325.3116	325.3112	+1.2	37.23	21:0	C_21_H_41_O_2_	0.18 ± 0.05
333.2802	333.2799	+0.9	24.08	22:3	C_22_H_37_O_2_	0.28 ± 0.04
335.2958	335.2956	+0.6	28.92	22:2	C_22_H_39_O_2_	0.39 ± 0.06
**337.3114**	**337.3112**	**+0.6**	**34.00**	**22:1**	**C_22_H_41_O_2_**	**3.5 ± 0.5**
**339.3266**	**339.3269**	**−0.9**	**40.41**	**22:0**	**C_22_H_43_O_2_**	**4.0 ± 0.9**
**353.3065**	**353.3061**	**+1.1**	**21.33**	**22:1;1**	**C_22_H_41_O_3_**	**2.6 ± 0.2**
353.3427	353.3425	+0.6	44.08	23:0	C_23_H_45_O_2_	0.27 ± 0.11
355.3226	355.3218	+2.3	16.32	22:0;1	C_22_H_43_O_3_	0.22 ± 0.10
361.3121	361.3112	+2.5	36.30	24:3	C_24_H_41_O_2_	0.25 ± 0.04
363.3275	363.3269	+1.7	39.18	24:2	C_24_H_43_O_2_	0.06 ± 0.01
365.3429	365.3425	+1.1	41.42	24:1	C_24_H_45_O_2_	0.20 ± 0.04
**367.3583**	**367.3582**	**+0.3**	**47.00**	**24:0**	**C_24_H_47_O_2_**	**1.2 ± 0.5**
383.3539	383.3531	+2.1	38.11	24:0;1	C_24_H_47_O_3_	0.09 ± 0.05

## Supplementary Materials

Figure S1. ESI(−)-FTMS spectra of a purified lipid extract of *L. luteus* seeds relevant to PA (A), PG (B), PI (C), PE (D), PC (E), and LPC (F) in a negative ion mode averaged under the corresponding chromatographic band (see Figure 1). Figure S2. Tandem MS spectra obtained by ESI(−)-CID of representative PA identified in the lipid extract of *L. luteus* seeds. Figure S3. Tandem MS spectra obtained by ESI(−)-CID of representative PG identified in the lipid extract of *L. luteus* seeds. Figure S4. Tandem MS spectra obtained by ESI(−)-CID of representative PI identified in the lipid extract of *L. luteus* seeds. Figure S5. Tandem MS spectra obtained by ESI(−)-CID of representative PE identified in the lipid extract of *L. Luteus* seeds. Figure S6. Tandem MS spectra obtained by ESI(-)-CID of representative PC identified in the lipid extract of *L. luteus* seeds. Figure S7. XIC chromatogram of a standard LPC 17:0/0:0 observed as a demethylated molecule at *m*/*z* 494.3 (A) and its relevant tandem MS spectrum (B). Figure S8. Tandem MS spectra obtained by ESI(−)-CID of representative LPC identified in the lipid extract of *L. luteus* seeds. Figure S9. XIC chromatogram for LPE 13:0/0:0 observed as a demethylated molecule at *m*/*z* 410.2 (A) and its relevant tandem MS spectrum (B).
