# Supplementary material for: Analysis of Phospholipids, Lysophospholipids, and Their Linked Fatty Acyl Chains in Yellow Lupin Seeds (Lupinus luteus L.) by Liquid Chromatography and Tandem Mass Spectrometry
Source: Molecules. 2020 Feb 13;25(4):805. doi: 10.3390/molecules25040805 (PMC7070507; doi:10.3390/molecules25040805)
Supplement: Supplementary file 1 [file molecules-25-00805-s001.pdf]

Analysis of phospholipids, lysophospholipids and their linked fatty acyl chains in yellow lupin seeds (Lupinus luteus L.) by liquid chromatography and tandem mass spectrometry

C.D. Calvano*[a,c], M. Bianco[b], G. Ventura[b], I. Losito [a,b], F. Palmisano[a,b], T.R.I. Cataldi[a,b]

[a]Centro Interdipartimentale SMART, [b]Dipartimento di Chimica, [c]Dipartimento di Farmacia-Scienze del Farmaco, Università degli Studi di Bari Aldo Moro, via Orabona 4, 70126 Bari (Italy)

Number of Figures: 9

Keywords: LC-ESI-tandem MS, food, phospholipids, fatty acids, *Lupinus luteus*.

*Author for correspondence, email: cosimadamiana.calvano@uniba.it, tommaso.cataldi@uniba.it

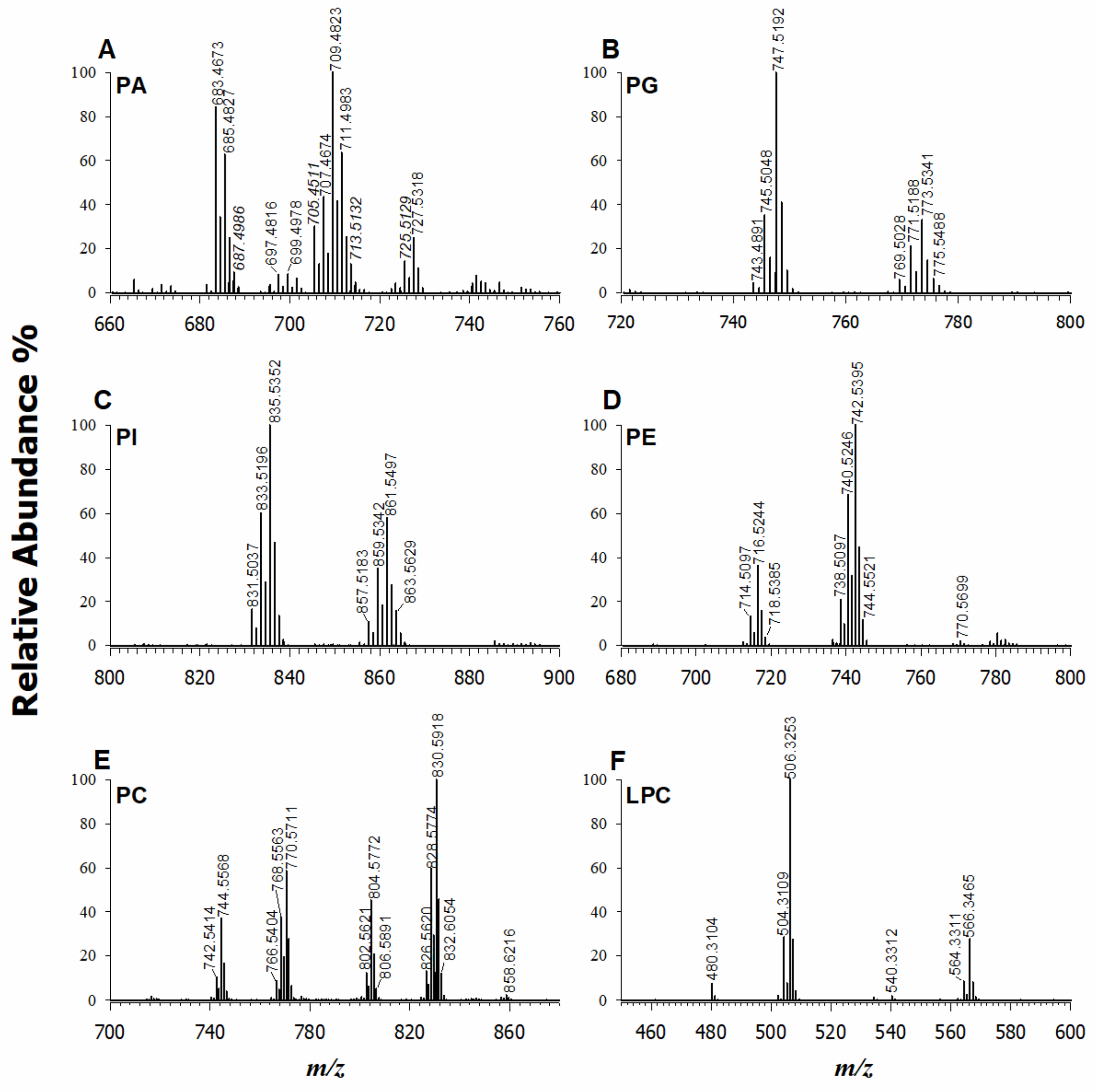

Figure S1.

Figure S1. ESI(-)-FTMS spectra of a purified lipid extract of *L. Luteus* seeds relevant to PA (A), PG (B), PI (C), PE (D), PC (E) and LPC (F) in negative ion mode averaged under the corresponding chromatographic band (see Figure 1).

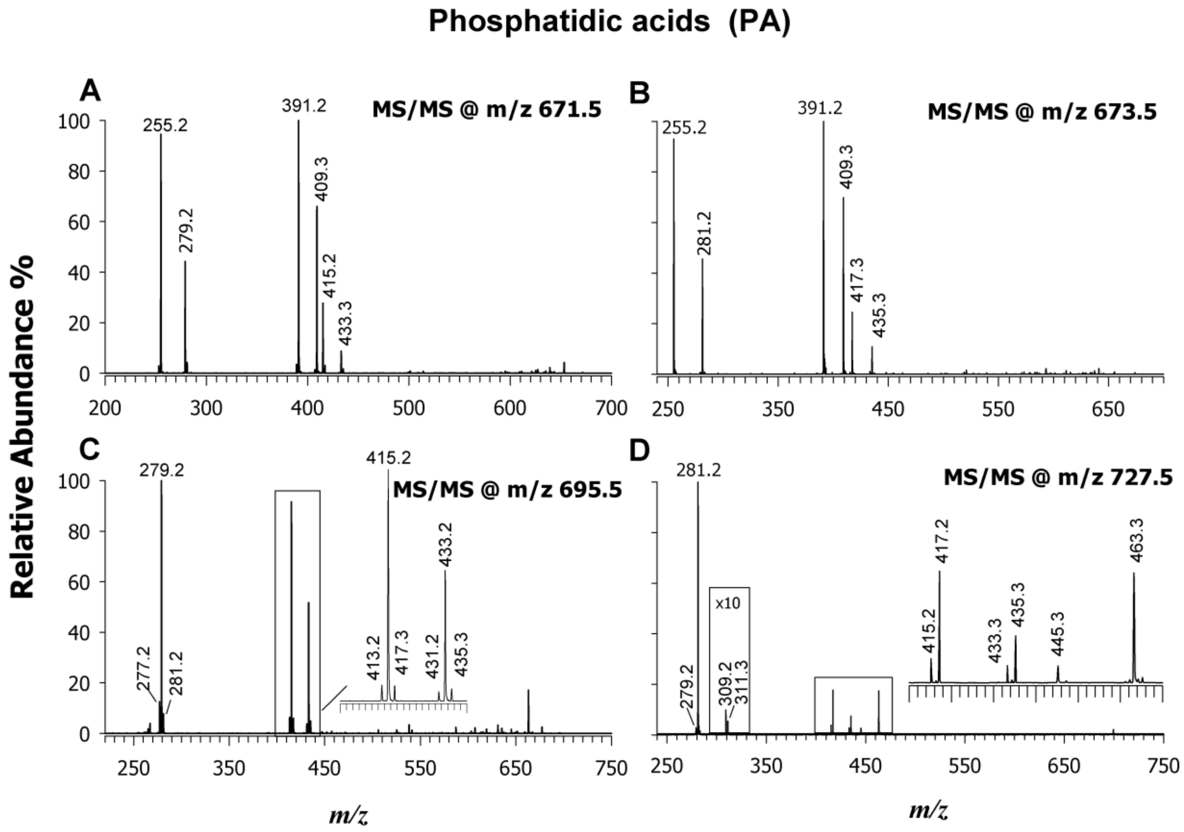

Figure S2. Tandem MS spectra obtained by ESI(-)-CID of representative PA identified in the lipid extract of *L. Luteus* seeds.

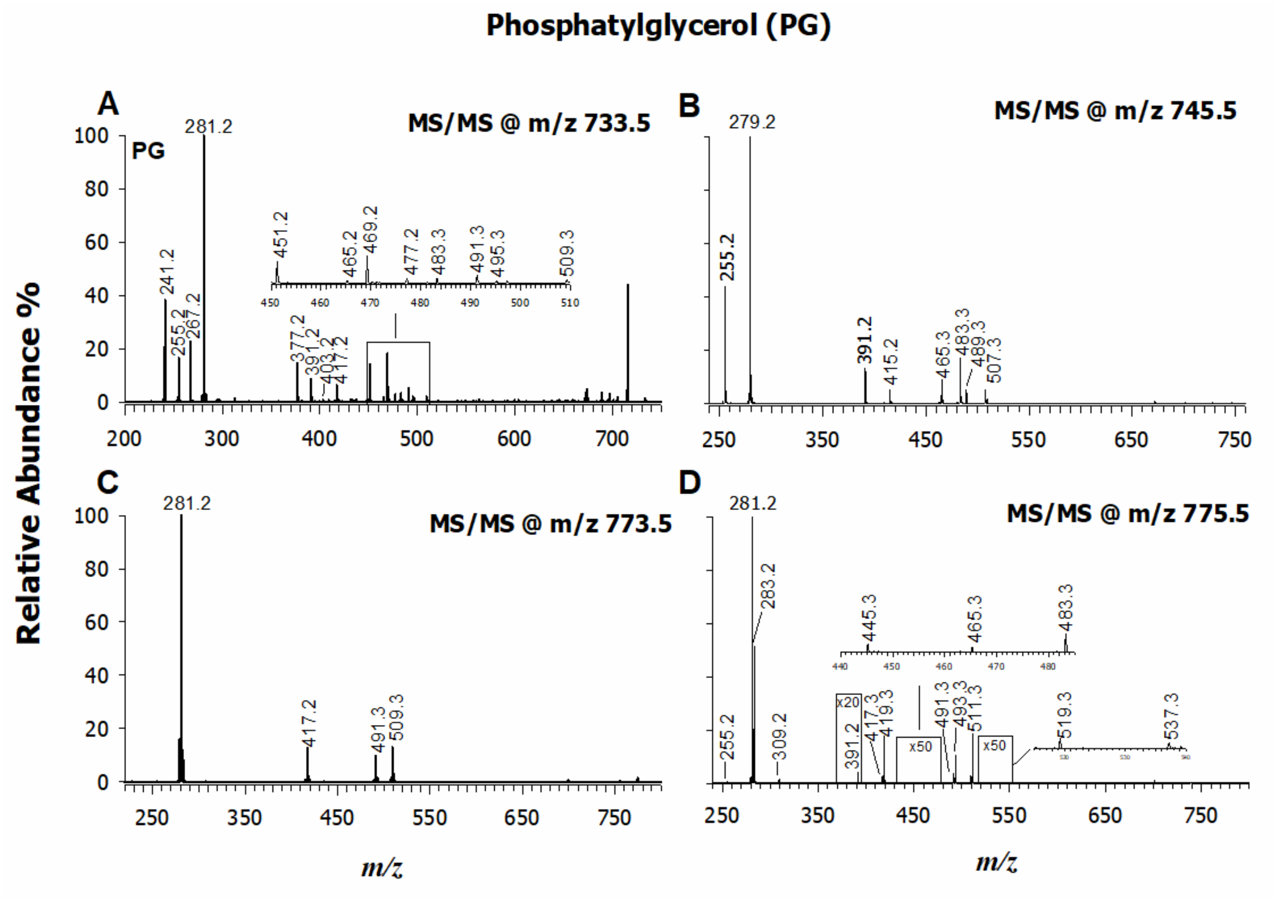

Figure S3. Tandem MS spectra obtained by ESI(-)-CID of representative PG identified in the lipid extract of *L. Luteus* seeds.

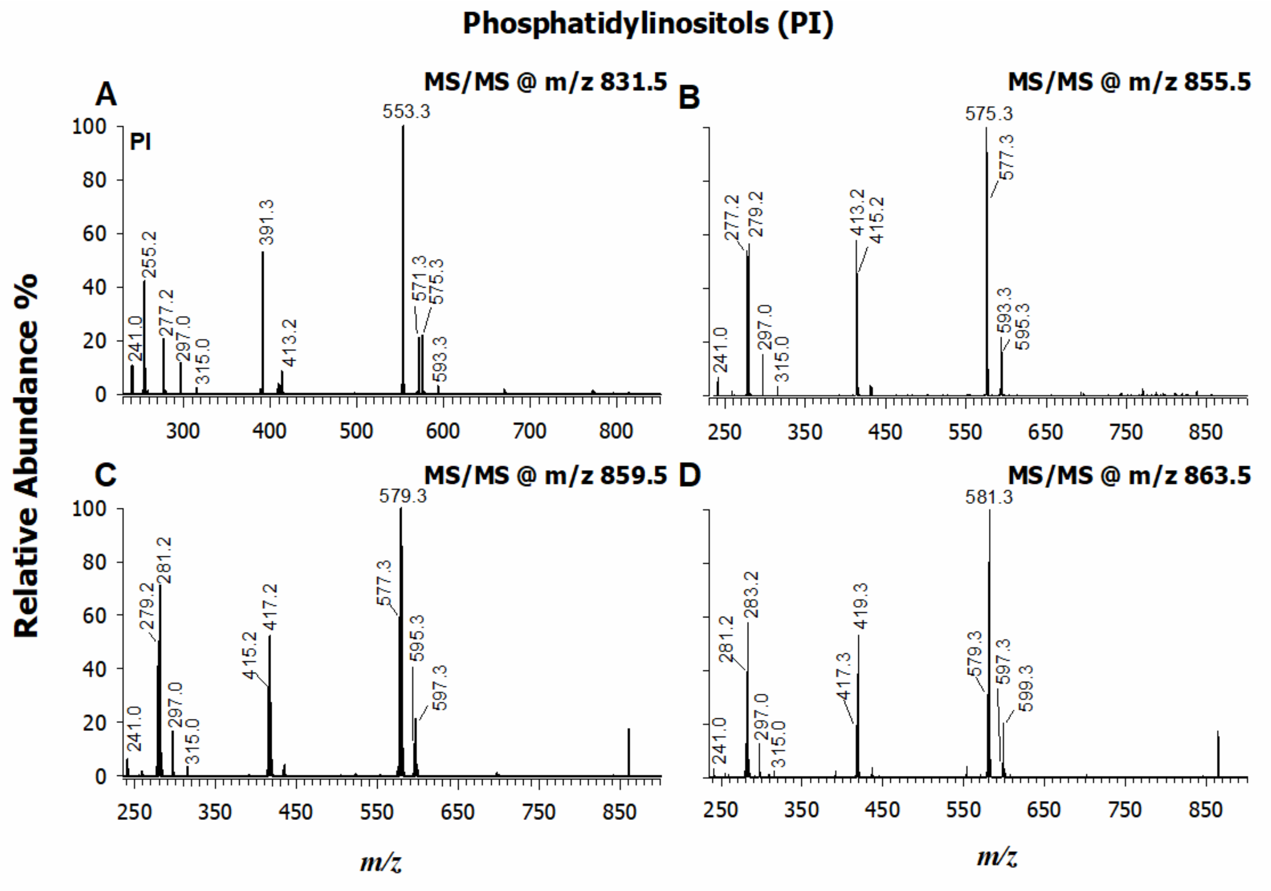

Figure S4. Tandem MS spectra obtained by ESI(-)-CID of representative PI identified in the lipid extract of *L. Luteus* seeds.

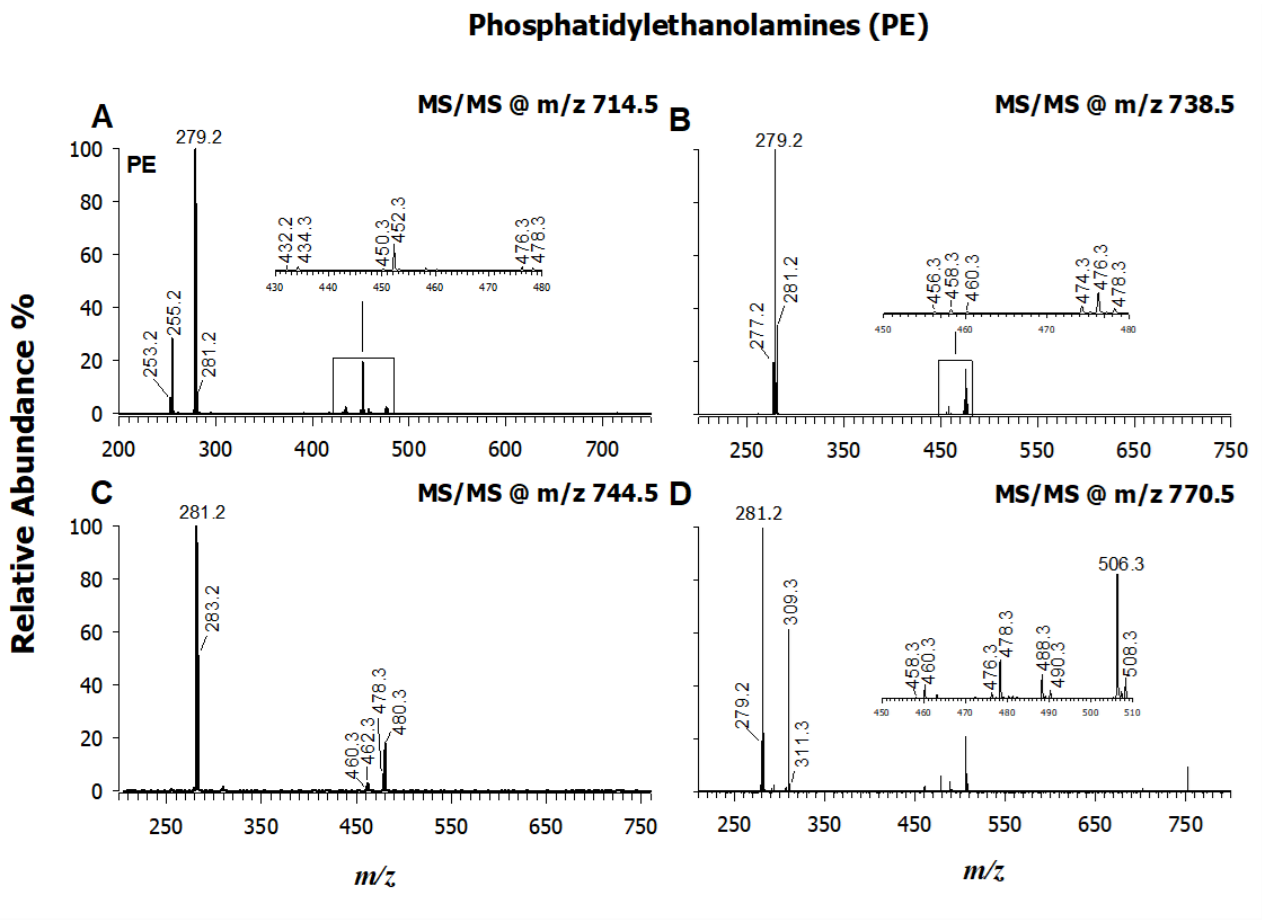

Figure S5. Tandem MS spectra obtained by ESI(-)-CID of representative PE identified in the lipid extract of *L. Luteus* seeds.

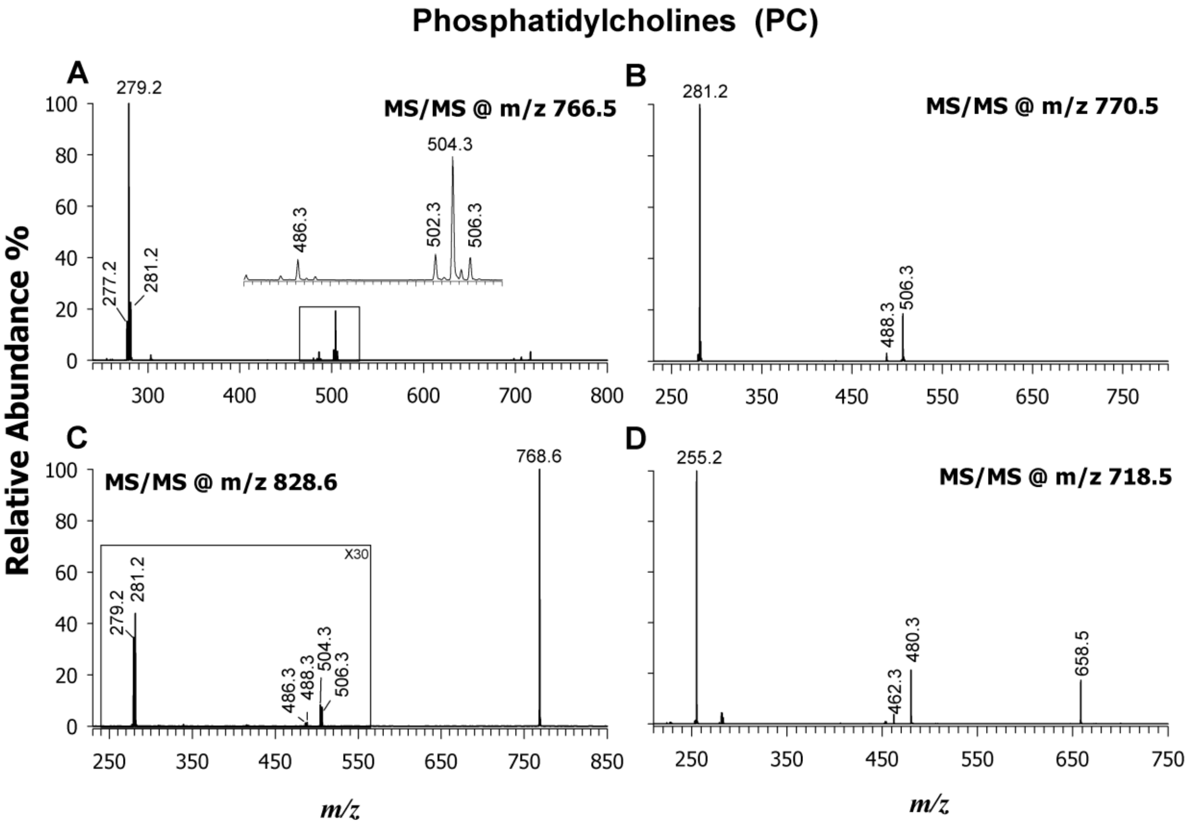

Figure S6. Tandem MS spectra obtained by ESI(-)-CID of representative PC identified in the lipid extract of *L. Luteus* seeds.

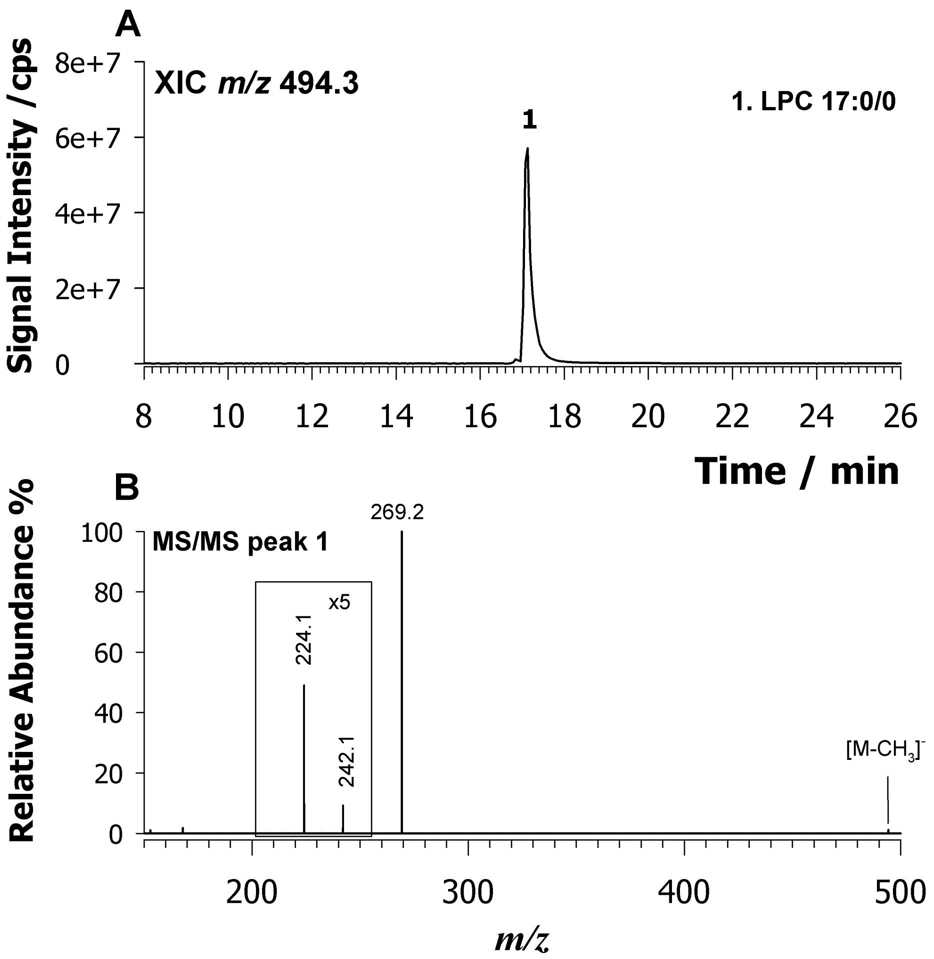

Figure S7.

Figure S7. XIC chromatogram of a standard LPC 17:0/0 observed as demethylated molecule at *m/z* 494.3 (A) and its relevant tandem MS spectrum (B).

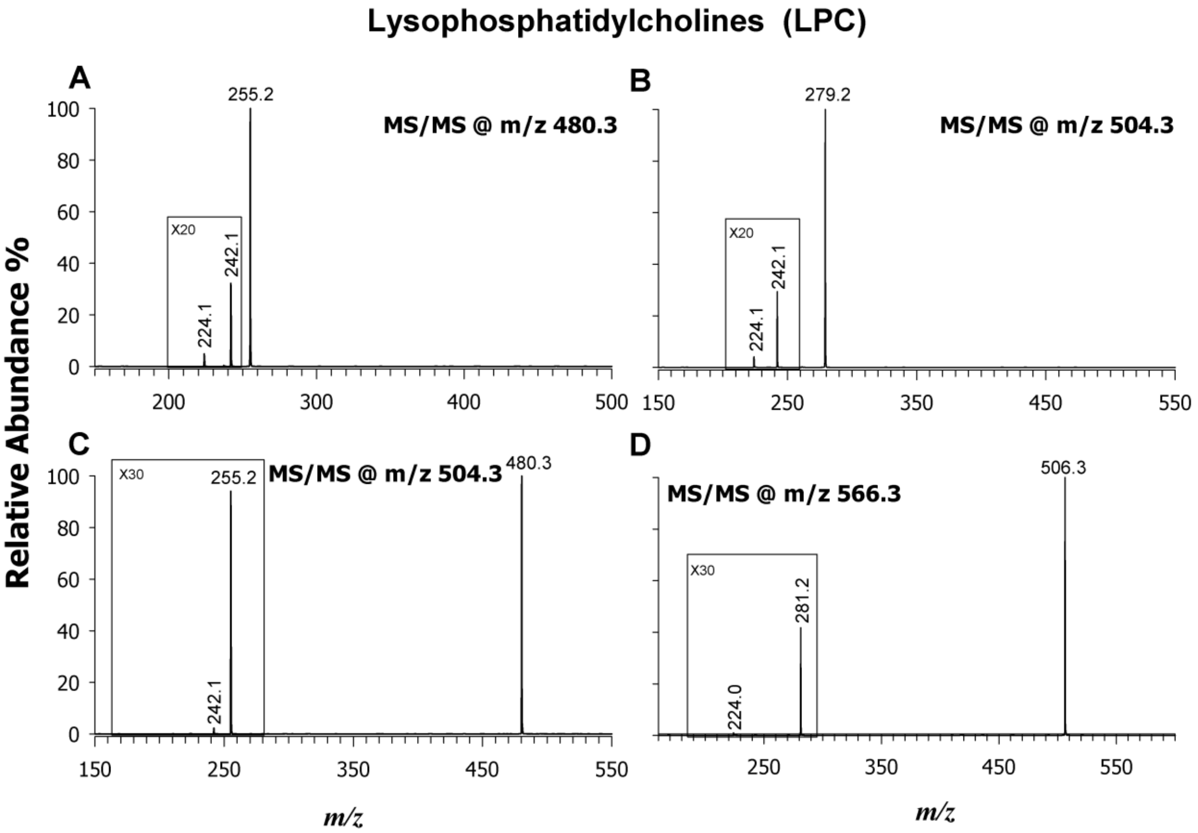

Figure S8. Tandem MS spectra obtained by ESI(-)-CID of representative LPC identified in the lipid extract of *L. Luteus* seeds.

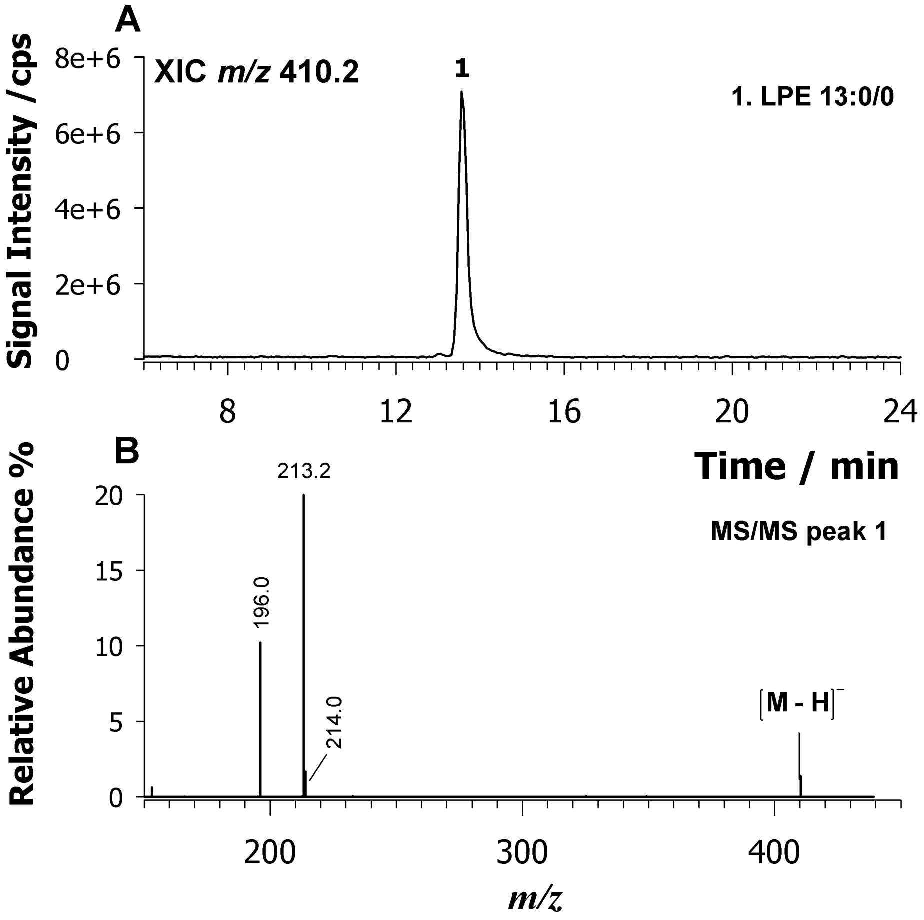

Figure S9. XIC chromatogram for LPE 13:0/0:0 observed as demethylated molecule at *m/z* 410.2 (A) and its relevant tandem MS spectrum (B).
